# An Indicator of environmental enrichment to measure physical, social and cognitive activities in human daily life

**DOI:** 10.1186/s12888-022-03952-w

**Published:** 2022-04-25

**Authors:** Mónica Flores-Ramos, María Yoldi-Negrete, Rodrigo Guiza-Zayas, Gerardo-Bernabé Ramírez-Rodríguez, Adolfo Montes-Castrejón, Ana Fresán

**Affiliations:** 1 Dirección de Enseñanza, Instituto Nacional de Psiquiatría “Ramón de la Fuente Muñiz”, Calzada México-Xochimilco 101, Col. San Lorenzo Huipulco, Tlalpan, C.P, 14370 Ciudad de México, Mexico; 2 Laboratorio de Epidemiología Clínica, Subdirección de Investigaciones Clínicas, Instituto Nacional de Psiquiatría “Ramón de la Fuente Muñiz”, Calzada México-Xochimilco 101, Col. San Lorenzo Huipulco, Tlalpan, C.P, 14370 Ciudad de México, Mexico; 3 Laboratorio de Neurogénesis, Subdirección de Investigaciones Clínicas, Instituto Nacional de Psiquiatría “Ramón de la Fuente Muñiz”, Calzada México-Xochimilco 101, Col. San Lorenzo Huipulco, Tlalpan, C.P, 14370 Ciudad de México, Mexico

**Keywords:** Environmental enrichment, Depression, Cognition, Physical activity, Social activity

## Abstract

**Background:**

The concept of environmental enrichment (EE) encompasses complex physical, social, cognitive, motor, and somatosensory stimuli to which individuals are differentially exposed. An indicator of EE comprising these elements would facilitate the study of the impact of EE in diverse clinical settings by allowing an easy and comparable measurement. This study aimed to create and test such an EE indicator based on the Florida Cognitive Activities Scale (FCAS), the Multidimensional Social Integration in Later Life Scale (SILLS), and the International Physical Activity Questionnaire (IPAQ).

**Methods:**

Participants with major depression and control subjects were recruited in this cross-sectional comparative study. Depressive symptom severity was assessed with the Hamilton Depression Rating Scale (HAM-D). The EE indicator was used to evaluate cognitive, social, and physical activity. We divided the sample into three levels of cognitive and social activities to construct an EE indicator and compared the obtained scores between participants with major depression and control subjects.

Results: 40 patients suffering from major depression and 50 control subjects were included. Higher HAM-D scores were associated with lower EE levels. Cognitive and social items exhibited adequate reliability. Control subjects reported higher scores in all three activities evaluated, except for some items of physical activities. This indicator of EE clearly differentiated between participants with major depression from control subjects.

**Conclusions:**

FCAS, SILLS, and IPAQ used together are valid to evaluate EE. This EE indicator may be a useful tool during clinical practice. The cross-sectional design and the small sample size are limitations of the present study.

## Background

Environmental enrichment (EE) is a model that combines complex physical, social, cognitive, motor, and somatosensory stimuli that have been proven to have multiple benefits in animals [1]. Metabolism, cognitive function, immunity, anxiety, and depression are improved by EE [2]. Their cognitive and behavioral effects seem to be mediated by several factors including growth factors, neurotransmitters, and neurotrophins such as the increased level of brain-derived neurotrophic factor (BDNF) in the hippocampus [1, 3, 4]; BDNF induction could also promote diverse biological changes in the metabolism, immunity, and suppression of cancer growth [2].

The benefits of the exposure to environmental stimuli and the consequences for brain function seen in animal models are likely to exist in humans, with many examples of the correlation between the presence of the components of poorly environmental enrichment and negative clinical consequences, i.e., the presence and impact on psychiatric disorders of poor social integration, lack of cognitive activity and diminished physical activity [5–11]. As such, depressed patients could be poorly interested in certain activities because of their symptoms [9–11], and they can serve as an example of low EE where an approximation for measuring EE could be tested.

Furthermore, the manipulation of environmental factors, alluding to the environmental enrichment provided in animal studies, seems to demonstrate similar benefic effects in clinical populations. Indeed, the exposure of humans to some of the elements of the EE have proven positive effects in psychiatric conditions, such as physical activity in depressed patients [12, 13], cognitive activities to reduce the risk of dementia [5, 14, 15] and social interaction for bipolar disorder [16]. Also, studies using a type of environmental enrichment for the rehabilitation after stroke are promising [17, 18], and some authors developed clinical EE adaptation to be also applied as a treatment for autism [19, 20] or as an adjuvant treatment for several disorders including schizophrenia, antisocial behavior, attention-deficit and hyperactivity [21].

However, the measurement of EE in humans is still controversial and the difficulties to translate this model to human life have been analyzed in some studies [22]. One of the difficulties is having a “standardized model” about EE, considering that housing conditions in animals among laboratories show differences in the complexity of EE, duration of the exposure to EE, the continuous or restricted exposure, thus making difficult its replication in rodents and its establishment for the human behavior [22–25]*.* In addition, it is yet not clear which components of the EE are the most important elements that benefit animals, although some reports have provided evidence of the benefits of cognitive, social, and physical activities [26–28].

An approximation to creating an instrument for measuring EE in humans could be to combine measures of the frequency of certain daily life activities related to the parameters of EE − such as social integration, cognitive activities, and physical activity− to create an integrated indicator of EE for clinical settings, which would, theoretically, adequately distinguish populations with different degrees of EE.

The aim of the present study was to validate an integrated indicator of EE obtained by combining the scores the Florida Cognitive Activities Scale (FCAS), the Multidimensional Social Integration in Later Life Scale (SILLS), and the International Physical Activity Questionnaire (IPAQ) (all adequate and useful instruments for the assessment of cognitive activities, social integration, and physical activity, respectively). For this purpose, we determined the validity and reliability of these three instruments, and secondly, we compared the scores obtained by combining these three instruments in an integrated indicator of EE (low, moderate, and high) between two populations with different degrees of EE: a sample of control subjects and a sample of participants with major depression, used as a proxy of low EE.

We expected an adequate validity and reliability of the integrated indicator of EE, as well as in the used instruments, with a significant correlation between them, and hypothesized that participants with major depression would show lower scores on all three instruments, as well as a lower score in the indicator of EE, in comparison to control subjects.

## Methods

### Study design and participants

This is a cross-sectional comparative study that involved the measurement of convergent validity and internal consistency of three scales and the creation of an EE indicator.

Participants with major depression (MD) were recruited from the outpatient service at a psychiatric facility in Mexico City which provides clinical psychiatric and psychological attention. They were diagnosed with major depressive disorder in accordance with the Diagnostic and Statistical Manual of Mental Disorders, fifth edition (DSM-5) criteria; with a minimum score of 13 in the 17- items Hamilton Depression Rating Scale (HDRS). All participants were free of any psychiatric medication. Control subjects (CS) were recruited from the general population using brochures placed into and around the hospital; they were interviewed by a clinical psychiatrist to verify inclusion criteria and discard the presence of any mental disorder; also, the Mini Neuropsychiatric Interview (MINI) was applied to discard psychiatric disorders in the CS. All participants were adults (> 18 years old) and after the aims and procedures of the study were fully explained, they gave their oral and written informed consent for their participation in the study guaranteeing the anonymity of their information, which was only be used for research purposes. The study was approved by the ethics committee of the National Institute of Psychiatry (CEI/C/041/2020).

### Assessment procedure

Demographic information such as gender, age, years of education, marital and occupational status was assessed with a face-to-face interview with all participants. For those with major depressive disorder, additional clinical information was obtained, such as the age of illness onset, illness evolution (years), current episode features (with anxious or melancholic symptoms), and duration in weeks.

The following three scales were adapted and used together for the assessment of the environmental enrichment and were answered by participants with major depression and by control subjects.

1. Florida Cognitive Activities Scale (FCAS). This scale comprises 25-self reported items that assess activities of different levels of cognitive effort (e.g. listen to music vs. write a text) performed during the last month answered on a 5-point Likert frequency scale (0 = never did this activity to 4 = do this activity every day). A total score is obtained by the sum of the individual item scores with higher scores reflecting a higher level of cognition. The scale has shown moderate values of reliability and validity with authors suggesting further cross-validation studies [29].
2. Multidimensional Social Integration in Later Life Scale (SILLS). This scale comprises 22-self-reported items assessing social integration in accordance with the performance of several activities during the last month (e.g., get together with the family, get together with friends, go to a gym, etc.). All items are scored on a 5-point Likert frequency scale (0 = never did this activity to 4 = do this activity every day). Eight additional items assessed satisfaction with social integration and are scored on a 5-point Likert satisfaction scale (0 = very unsatisfied to 4 = very satisfied). Therefore, two separate scores (social integration and satisfaction) and a total SILLS score (the sum of social integration and satisfaction scores) can be obtained. Higher scores reflect higher social integration. In the study where the scale was developed, the overall internal consistency of the scale was adequate (Cronbach’s alpha = 0.86) [30].
3. International Physical Activity Questionnaire (IPAQ). The short version of 7 items of the IPAQ was used for the present study. Items assessed the frequency (days per week), intensity (intense and moderate activities, walking and sitting), and duration (all assessed in minutes per day except for sitting which was evaluated by hours per day) of physical activities. This questionnaire has become one of the most widely used physical activity questionnaires and the short version is mostly used for surveillance purposes in several countries [31–33]. Weekly activity is reported in metabolic equivalent of task (MET) and three main categories of activity (low, moderate, high) can also be determined [34]. Moderate reliability and validity values have been reported in the Mexican adult population [35], although its utility for surveillance purposes has been continuously reported.

In addition to the assessment of parameters related to EE with these scales, depressive symptom severity was assessed in all participants with the 17-item Hamilton Depression Rating Scale (HAM-D), one of the widest clinician-administered depression scales [36] with adequate concurrent validity values for its use in Mexican population [37].

### Statistical analysis

For sample description, frequencies and percentages for categorical variables and means and standard deviations (S.D.) for continuous variables were used. Demographic features were compared between groups using chi-square (χ^2^) tests for categorical variables and independent-sample t-tests for the comparison of continuous variables.

To evaluate convergent validity of the scales assessing the environment enrichment, Pearson correlation coefficients of the total scores of the FCAS, SILLS, and IPAQ with the total score of the HAM-D were determined as well as the correlation between the three environmental enrichment scales. Reliability of the FCAS, SILLS, and IPAQ scales was determined with Cronbach’s alpha, with acceptable reliability values equal to or greater than 0.75. To test the hypothesis of a lower environmental enrichment in participants with major depression, independent samples t-tests were used to compare the total scores of the FCAS, SILLS, and IPAQ between participants with depression and control subjects.

To determine the EE indicator, we used the scores of the FCAS and SILLS of control subjects, dividing the scores into three main levels (low, moderate, and high) according to percentile 33.3 and 66.6, while these levels are already defined for the IPAQ. We define low, moderate, and high EE according to the following definitions:

- *Low EE*: 2 or 3 of the cognitive, social, and physical dimensions identified in the low category.
- *Moderate EE*: 2 or 3 of the cognitive, social, and physical dimensions identified in the moderate category or one category identified as low, another as moderate, and the third one as high.
- *High EE:* 2 or 3 of the cognitive, social, and physical dimensions identified in the high category.

In accordance with these definitions, the EE levels were described and compared between participants with depression and control subjects.

All analyses were performed with the SPSS 21.0 software for Windows and the significance level for tests was established at *p* < 0.05.

## Results

### Sample description

A total of 90 participants were included for the study, 40 (44.4%) were participants with major depression and 50 (55.6%), control subjects. Most of the participants were women and single in both groups, with similar age at the time of the study. CS reported more years of education than MD. A higher number of MD participants were unemployed or dedicated to housewife activities (See Table 1).

**Table 1:** Demographic features between CS and MD participants

	Control subjects *n* = 50	Major depression *n* = 40	Statistic
Gender – Women *n %*	35 70	33 82.5	χ2 = 1.8, df 1, *p* = 0.17
Age (years) *Mean S.D*.	30.1 11.0	31.0 10.7	t = −0.3, df 88, *p* = 0.69
Marital status - single *n %*	39 78.0	31 77.5	χ2 = 0.6, df 2, *p* = 0.71
Education (years) *Mean S.D.*	16.0 3.8	14.1 3.1	t = 2.5, df 88, *p* = 0.01
Occupation n %			
Unemployed	2 4.0	8 20.0	
Housewife activities	1 2.0	4 10.0	χ2 = 9.2, df 3, *p* = 0.02
Students	33 46.0	13 32.5	
Remunerated activity	24 48.0	15 37.5	

For MD participants, the age of illness onset was at 21.2 (S.D. = 8.9 years – reported by 38 participants) with a total illness evolution of 8.9 years (S.D. = 8.5). The mean duration of the current episode was 26.2 (S.D. = 28.2) weeks. The total score of the Hamilton Depression Rating Scale was higher in MD participants than in CS (22.8, S.D. 5.3 vs. 3.9, S.D. = 2.8, t = −21.5, 88 df, *p* < .001) reflecting severe depression in the first group [38].

### Convergent validity and internal consistency of the EE indicator

To obtain the convergent validity of the scale, we used the total score of the Hamilton Depression Rating Scale. As seen in Table 2, the correlation analysis showed that higher depressive symptom severity is associated with lower levels of cognition (FCAS score), lower social integration, satisfaction, and global social integration (SILLS total scores), and lower weekly activity reported in metabolic equivalent of task (IPAQ total score).

**Table 2:** Convergent validity of the enriched environment scales – Pearson correlations with the HAM-D total score

Enriched environment scales	Ham-D Total score r (p)
Florida Cognitive Scale (FCAS total score)	−0.48 (< 0.001)
Multidimensional Social Integration in Later Life Scale (SILLS)	
Social integration dimension	−0.53 (< 0.001)
Satisfaction dimension	−0.43 (< 0.001)
Total score	−0.55 (< 0.001)
International Physical Activity Questionnaire (IPAC total score)	−0.39 (< 0.001)

Internal consistency of the scale was obtained for the total sample and CS participants and MD participants. All the scales, except for the IPAQ exhibited adequate Cronbach’s alpha values (see Table 3).

**Table 3:** Internal consistency of the enriched environment scales: total sample, CS participants and MD participants

	Total sample *n* = 90	Control subjects *n* = 50	Major depression *n* = 40
Florida Cognitive Scale (FCAS)	0.85	0.78	0.84
Multidimensional Social Integration Scale (SILLS)			
Social integration dimension	0.91	0.88	0.88
Satisfaction dimension	0.86	0.80	0.86
Total score	0.92	0.88	0.91
International Physical Activity Questionnaire (IPAC)	0.23	0.07	0.29

### Comparison of scale results between control subjects and participants with major depression

Due to the low reliability obtained from the IPAQ, we decided to perform the comparison between groups of each of the IPAQ items and the total score. In general, CS reported higher scores in all items, except for item 5 “days per week walking”, item 6 “minutes of walking per day”, and item 7 “hours sitting in a business day in the last week”, where both groups display similar scores (see Table 4).

**Table 4:** Comparative analysis of the enriched environment scales: Data from CS participants and MD participants

	Control subjects *n* = 50	Major depression *n* = 40	Statistic
	Mean (S.D.)		
Florida Cognitive Scale (FCAS)	47.9 (11.1)	34.8 (12.5)	t = 5.2, *p* < 0.001
Multidimensional Social Integration Scale (SILLS)			
Social integration dimension	60.2 (12.8)	45.4 (11.4)	t = 5.6, *p* < 0.001
Satisfaction dimension	28.5 (5.0)	22.6 (6.3)	t = 4.8, *p* < 0.001
Total score	88.7 (15.1)	68.1 (15.8)	t = 6.2, *p* < 0.001
International Physical Activity Questionnaire (IPAQ)			
Intense activities - days per week	3.0 (2.2)	1.3 (2.0)	t = 3.6, *p* < 0.001
Intense activities - minutes per day	41.3 (34.9)	24.2 (42.7)	t = 2.0, *p* = 0.04
Moderate activities - days per week	2.4 (2.1)	1.3 (1.7)	t = 2.6, *p* = 0.01
Moderate activities - minutes per day	35.7 (39.5)	17.0 (25.0)	t = 2.5, *p* = 0.01
Walking - days per week	4.8 (2.2)	4.1 (2.4)	t = 1.3, *p* = 0.17
Walking - minutes per day	40.7 (35.2)	32.5 (31.2)	t = 1.1, *p* = 0.24
Sitting – hours in a business day in the last week	7.1 (3.6)	7.3 (3.2)	t = −0.2, *p* = 0.78
Total score (MET)	2559.3 (1958.9)	1345.0 (1334.7)	t = 3.3, *p* = 0.001

### Environmental enrichment Indicator

The percentiles for dividing the sample into three levels for the FCAS (cognitive activities) were as follows: Low cognitive activities (score from 0 to 42), moderate cognitive activities (score from 43 to 56), and high cognitive activities (score from 57 onwards). For social integration (SILLS scale) levels were defined as follows: Low social integration (score from 0 to 78), moderate social integration (score from 79 to 98), and high social integration (score from 99 onwards).

The comparison between control subjects and participants with major depression showed that the latter exhibited lower levels of cognitive activities, social integration, and physical activities. We highlighted that 72.5% of participants with major depression report a low environmental enrichment when compared to the 24.0% reported by control subjects (see Table 5).

**Table 5:** Levels of cognitive activities, social integration, physical activity, and level of environmental enrichment

	Total sample *n* = 90	Control Subjects *n* = 50	Major depression *n* = 40	Statistics
Level of cognitive activities *n %*				
Low	48 53.3	17 34.0	31 77.5	
Moderate	25 27.8	19 38.0	6 15.0	χ^2^ = 17.0, *p* < 0.001
High	17 18.9	14 28.0	3 7.5	
Level of social integration *n %*				
Low	48 53.3	17 34.0	31 77.5	
Moderate	23 25.6	16 32.0	7 17.5	χ^2^ = 18.5, *p* < 0.001
High	19 21.1	17 34.0	2 5.0	
Level of physical activities *n %*				
Low	22 24.5	8 16.0	14 35.0	
Moderate	28 31.1	11 22.0	17 42.5	χ^2^ = 14.0, *p* = 0.001
High	40 44.4	31 62.0	9 22.5	
Level of environmental enrichment *n %*				
Low	41 45.6	12 24.0	29 72.5	
Moderate	29 32.2	20 40.0	9 22.5	χ^2^ = 23.1, *p* < 0.001
High	20 22.2	18 36.0	2 5.0	

## Discussion

Environmental enrichment is a broad concept that has been studied predominantly in animals, generating promising evidence for the prevention and treatment of mental disorders. Therefore, translational research on humans could be an important opportunity to evaluate the effects of the main EE components in human beings. Thus, our main objective was to create an indicator to measure EE based on cognitive, social, and physical activities performed daily, for clinical populations.

We observed that the internal consistency of the FCAS and SILLS was high as reflected by a Cronbach’s alpha > 0.8. However, the IPAQ had a low internal consistency, a finding coherent with other studies including a meta-analysis to validate IPAQ which reported high variability in the studies and lower correlation with objective measures of physical activities [39]. These differences could be derived from the evaluation of moderate-to-vigorous physical activity (MVPA) and the sedentary life. However, our results support its usefulness for discriminating subjects with less physical activity (patients with depression) when compared to control subjects and, along with the cognitive and social instruments, showed an inverse correlation with the HAM-D scores, confirming the validity of the instruments [40].

The comparison between participants with major depression and control subjects confirmed our hypothesis: this indicator clearly distinguishes between depressed subjects and controls, a finding that renders this EE indicator a promising tool for clinical practice and EE research.

The most important limitation of the present study is the sample size, particularly as three different levels of environmental enrichment were established and analyzed, additional research should include larger samples sizes to avoid biases and support or not our results. Another limitation is the low generalizability of our findings as the recruitment was performed in a single location, and comparisons were performed with only one clinical entity. Also, the cut-off points obtained from the SILLS and FCAS scales were determined based on statistical procedures and not by sensitivity and sensibility analysis, which should be performed in future studies using other procedures. In addition, self-reporting of social integration and cognitive effort should be taken with caution as participants may include other problems not related to what the scales evaluate, may under-or over qualify these features, or may even be affected by social desirability. Other studies could investigate whether different approximations to measuring physical activity would further improve the accuracy of this EE indicator for detecting low EE environments. One last concern is the use of self-reported measures to evaluate EE, as.

Despite these important limitations, the relevance of the present study relies on the creation of an EE indicator that can be used to translate the investigations of the fascinating field of EE to humans. Thus, our study points to the direction of the relevance of the exposure to environmental stimuli to prevent depression. In this line, a previous study performed in rodents suggests that EE provided by cognitive, social, and physical stimuli prevent the development of depression-like behavior [41]. Then, the results of our study reflect an early approximation in the long road searching for an accurate evaluation of daily activities related to the so-called environmental enrichment in humans. The importance of an accurate assessment of these variables is now undeniable, as the aim of clinical treatment evolves from mere symptomatic recovery to an improvement in the quality of life as is suggested by studies done in animal models [42, 43].

## Conclusion

We consider that the proposed EE indicator is valid to be used in clinical settings and allows us to evaluate the grade of EE according to daily activities in healthy people and depressed patients.

## Data Availability

The information analyzed in the current study is not publicly available due to the confidentiality of the participants but is available from the corresponding author on request.

## Abbreviations

CS: Control subjects
EE: Environmental enrichment
FCAS: Florida Cognitive Activities Scale
IPAQ: International Physical Activity Questionnaire
MD: Major depression
MET: Metabolic equivalent of task
MVPA: Moderate-to-vigorous physical activity
SILLS: Multidimensional Social Integration in Later Life Scale

## References

[1] Nithianantharajah J, Hannan AJ (2006). Enriched environments, experience-dependent plasticity and disorders of the nervous system. Nat Rev Neurosci.

[2] Queen NJ, Hassan QN, Cao L (2020). Improvements to healthspan through environmental enrichment and lifestyle interventions: where are we now?. Front Neurosci.

[3] Jha S, Dong B, Sakata K (2011). Enriched environment treatment reverses depression-like behavior and restores reduced hippocampal neurogenesis and protein levels of brain-derived neurotrophic factor in mice lacking its expression through promoter IV. Transl Psychiatry.

[4] Ramírez-Rodríguez G, Ocaña-Fernández MA, Vega-Rivera NM, Torres-Pérez OM, Gómez-Sánchez A, Estrada-Camarena E, Ortiz-López L (2014). Environmental enrichment induces neuroplastic changes in middle age female Balb/c mice and increases the hippocampal levels of BDNF, p-Akt and p-MAPK1/2. Neuroscience.

[5] Gaino LV, de Almeida LY, de Oliveira JL, Nievas AF, Saint-Arnault D, de Souza JJ (2019). The role of social support in the psychological illness of women. Rev Lat Am Enfermagem.

[6] Sattler C, Toro P, Schönknecht P, Schröder J (2012). Cognitive activity, education and socioeconomic status as preventive factors for mild cognitive impairment and Alzheimer's disease. Psychiatry Res.

[7] Vaughan L, Erickson KI, Espeland MA, Smith JC, Tindle HA, Rapp SR (2014). Concurrent and longitudinal relationships between cognitive activity, cognitive performance, and brain volume in older adult women. J Gerontol B Psychol Sci Soc Sci.

[8] Wilson RS, Bennett DA, Bienias JL, De Leon CM, Morris MC, Evans DA (2003). Cognitive activity and cognitive decline in a biracial community population. Neurology.

[9] Godard J, Grondin S, Baruch P, Lafleur MF (2011). Psychosocial and neurocognitive profiles in depressed patients with major depressive disorder and bipolar disorder. Psychiatry Res.

[10] Rock PL, Roiser JP, Riedel WJ, Blackwell AD (2014). Cognitive impairment in depression: a systematic review and meta-analysis. Psychol Med.

[11] Sumiyoshi T, Watanabe K, Noto S, Sakamoto S, Moriguchi Y, Tan KHX, Hammer-Helmich L, Fernandez J (2019). Relationship of cognitive impairment with depressive symptoms and psychosocial function in patients with major depressive disorder: cross-sectional analysis of baseline data from PERFORM-J. J Affect Disord.

[12] Craft LL, Perna FM (2004). The benefits of exercise for the clinically depressed. Rim care companion. J Clin Psychiatry.

[13] Morres ID, Hatzigeorgiadis A, Stathi A, Comoutos N, Arpin-Cribbie C, Krommidas C, Theodorakis Y (2019). Aerobic exercise for adult patients with major depressive disorder in mental health services: a systematic review and meta-analysis. Depress Anxiety.

[14] Najar J, Östling S, Gudmundsson P, Sundh V, Johansson L, Kern S, Guo X, Hällström T, Skoog I (2019). Cognitive and physical activity and dementia: a 44-year longitudinal population study of women. Neurology.

[15] Stanton R, Reaburn P (2014). Exercise and the treatment of depression: a review of the exercise program variables. J Sci Med Sport.

[16] Frank E, Swartz HA, Boland E (2007). Interpersonal and social rhythm therapy: an intervention addressing rhythm dysregulation in bipolar disorder. Dialogues Clin Neurosci.

[17] Janssen H, Ada L, Bernhardt J, McElduff P, Pollack M, Nilsson M, Spratt NJ (2014). An enriched environment increases activity in stroke patients undergoing rehabilitation in a mixed rehabilitation unit: a pilot non-randomized controlled trial. Disabil Rehabil.

[18] White JH, Bartley E, Janssen H, Jordan LA, Spratt N (2015). Exploring stroke survivor experience of participation in an enriched environment: a qualitative study. Disabil Rehabil.

[19] Aronoff E, Hillyer R, Leon M (2016). Environmental enrichment therapy for autism: outcomes with increased access. Neural Plast.

[20] Woo CC, Leon M (2013). Environmental enrichment as an effective treatment for autism: a randomized controlled trial. Behav Neurosci.

[21] Raine A, Mellingen K, Liu J, Venables P, Mednick SA (2003). Effects of environmental enrichment at ages 3-5 years on schizotypal personality and antisocial behavior at ages 17 and 23 years. Am J Psychiatry.

[22] Kempermann G (2019). Environmental enrichment, new neurons and the neurobiology of individuality. Nat Rev Neurosci.

[23] Rojas-Carvajal M, Sequeira-Cordero A, Brenes JC (2020). Neurobehavioral effects of restricted and unpredictable environmental enrichment in rats. Front Pharmacol.

[24] Rosenzweig MR, Bennett EL, Diamond M (1967). Effects of differential environments on brain anatomy and brain chemistry. Proc Annu Meet Am Psychopathol Assoc.

[25] Widman DR, Rosellini RA (1990). Restricted daily exposure to environmental enrichment increases the diversity of exploration. Physiol Behav.

[26] Fabel K, Wolf S, Ehninger D, Babu H, Galicia P, Kempermann G (2009). Additive effects of physical exercise and environmental enrichment on adult hippocampal neurogenesis in mice. Front Neurosci.

[27] Grégoire CA, Bonenfant D, Le Nguyen A, Aumont A, Fernandes KJ (2014). Untangling the influences of voluntary running, environmental complexity, social housing and stress on adult hippocampal neurogenesis. PLoS One.

[28] Moreno-Jiménez EP, Jurado-Arjona J, Ávila J, Llorens-Martín M (2019). The social component of environmental enrichment is a pro-neurogenic stimulus in adult c57BL6 female mice. Front Cell Dev Biol.

[29] Schinka JA, McBride A, Vanderploeg RD, Tennyson K, Borenstein AR, Mortimer JA (2005). Florida cognitive activities scale: initial development and validation. J Int Neuropsychol Soc.

[30] Fuller-Iglesias HR, Rajbhandari S (2016). Development of a multidimensional scale of social integration in later life. Res Aging.

[31] Craig CL, Marshall AL, Sjöström M, Bauman AE, Booth ML, Ainsworth BE, Pratt M, Ekelund U, Yngve A, Sallis JF, Oja P (2003). International physical activity questionnaire: 12-country reliability and validity. Med Sci Sports Exerc.

[32] Ekelund U, Sepp H, Brage S, Becker W, Jakes R, Hennings M, Wareham NJ (2006). Criterion-related validity of the last 7-day, short form of the international physical activity questionnaire in Swedish adults. Public Health Nutr.

[33] Wolin KY, Heil DP, Askew S, Matthews CE, Bennett GG (2008). Validation of the international physical activity questionnaire-short among blacks. J Phys Act Health.

[34] Carrera Y (2017). Cuestionario Internacional de actividad física (IPAQ). Enfermería del Trabajo.

[35] Medina C, Barquera S, Janssen I (2013). Validity and reliability of the international physical activity questionnaire among adults in Mexico. Rev Panam Salud Publica.

[36] Hamilton M (1967). Development of a rating scale for primary depressive illness. Br J Soc Clin Psychol.

[37] Berlanga C, Cortés J, Bauer J (1992). Adaptación y validación de la Escala de Depresión de Carroll en español. Salud Ment.

[38] National Institute for Clinical Excellence (2004). Depression: management of depression in primary and secondary care.

[39] Lee PH, Macfarlane DJ, Lam TH, Stewart SM (2011). Validity of the international physical activity questionnaire short form (IPAQ-SF): a systematic review. Int J Behav Nutr Phys Act.

[40] Culpepper L (2015). Impact of untreated major depressive disorder on cognition and daily function. J Clin Psychiatry.

[41] Vega-Rivera NM, Ortiz-Lopez L, Gomez-Sanchez A, Oikawa-Sala J, Estrada-Camarena EM, Ramírez-Rodríguez G (2016). The neurogenic effects of an enriched environment and its protection against the behavioral consequences of chronic mild stress persistent after enrichment cessation in si-month-old female Balb/C mice. Behav Brain Res.

[42] Branchi I, Santarelli S, Capoccia S, Poggini S, D’Andrea I, Cirulli F, Alleva E (2013). Antidepressant treatment outcome depends on the quality of the living environment: a pre-clinical investigation in mice. PLoS One.

[43] Ramírez-Rodríguez G, Vega-Rivera NM, Meneses-San Juan D, Ortiz-López L, Estrada-Camarena EM, Flores-Ramos M (2021). Short daily exposure to environmental enrichment, fluoxetine, or their combination reverses deterioration of the coat and anhedonia behaviors with differential effects on hippocampal neurogenesis in chronically stressed mice. Int J Mol Sci.

